# Node Interference and Robustness: Performing Virtual Knock-Out Experiments on Biological Networks: The Case of Leukocyte Integrin Activation Network

**DOI:** 10.1371/journal.pone.0088938

**Published:** 2014-02-20

**Authors:** Giovanni Scardoni, Alessio Montresor, Gabriele Tosadori, Carlo Laudanna

**Affiliations:** 1 Center for BioMedical Computing (CBMC), University of Verona, Verona, Italy; 2 Department of Pathology and Diagnostic, University of Verona, Verona, Italy; Umeå University, Sweden

## Abstract

The increasing availability of large network datasets derived from high-throughput experiments requires the development of tools to extract relevant information from biological networks, and the development of computational methods capable of detecting qualitative and quantitative changes in the topological properties of biological networks is of critical relevance. We introduce the notions of node 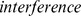 and 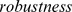 as measures of the reciprocal influence between nodes within a network. We examine the theoretical significance of these new, centrality-based, measures by characterizing the topological relationships between nodes and groups of nodes. Node interference analysis allows topologically determining the context of functional influence of single nodes. Conversely, the node robustness analysis allows topologically identifying the nodes having the highest functional influence on a specific node. A new Cytoscape plug-in calculating these measures was developed and applied to a protein-protein interaction network specifically regulating integrin activation in human primary leukocytes. Notably, the functional effects of compounds inhibiting important protein kinases, such as SRC, HCK, FGR and JAK2, are predicted by the interference and robustness analysis, are in agreement with previous studies and are confirmed by laboratory experiments. The interference and robustness notions can be applied to a variety of different contexts, including, for instance, the identification of potential side effects of drugs or the characterization of the consequences of genes deletion, duplication or of proteins degradation, opening new perspectives in biological network analysis.

## Introduction

Study of complex networks currently spans several disciplines, including biology, pharmacology, economy, social science, computer science and physics [1]. One of the major goals of modern network science is the quantitative characterization of network structure and functionality with the purpose of inferring emergent properties of complex systems, abstracted as networks and represented as graphs [2]. The topological analysis approach allows understanding the functionality of networks through the analysis of their specific architecture. For instance, the topological structure of a road network affects critical traffic jam areas, the topology of social networks affects the spread of information or diseases, and the topology of power grids affects the robustness and stability of the energy distribution [3]. Thus, few unifying principles, underlying the topology of networks, span different fields of science [4]; [5]; [6]; [7]; [8]; [9]; [10]. Remarkable results have been obtained in the field of biological network analysis, either in case of gene, protein or metabolite networks, and, even if far from being completely unveiled, several key notions have been introduced. In this context, indexes of network centrality such as degree, eccentricity, closeness, betweenness, stress, centroid and radiality [9], [10], [11] are topological parameters allowing quantifying the topological relevance of single nodes in a network. To date, network analysis is mainly focused on global network properties and on their global modifications [12]; [13]; [14]; [15] as in the case of the vitality index [9] or attack tolerance of networks [16]. Recent fundamental results [17] show how analysis on the topology of the network allows identifying the driving nodes of a network, i.e. the nodes that have to be controlled in order to control the entire network, suggesting that identification of these nodes depends on the network topology and not on the network dynamics. These results may suggest the utility of a deeper analysis of biological networks, with the purpose of analyzing not only global network properties, but especially local properties affecting those nodes that are, more than others, central to the global functionality of the network. In this study we introduce the notions of node 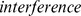 and 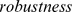 to characterize the domain of influence of single nodes. The interference notion applies the same principle of the “variable interference” used in security for computer programs [18]. It consists on changing the starting value of a single target variable and evaluating the changes on the other program variables during the computation: those variables showing greater changes are the set of program variables more dependent on the target variable. The node 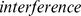 notion applies the same principle, based on the general perspective of a virtual knock-out experiment which can be summerized as follow: a node is removed from the network and the effects of such removal on the network structure are analyzed. In a node-centered perspective, centralities are the right parameters to evaluate in order to detect the effects of a single node alteration. As the centrality value of a node is strictly dependent on the network structure and on the properties of other nodes in the network, the consequences of a node deletion are well captured by the variation on the centrality values of all the other nodes. Notably, this kind of approach can model common situations where nodes are really removed or added from/to a physical network. In some cases, such as in social and financial networks, the structure of the network is naturally modified over time; in other cases this can be due to specific network changes: power grid failures, traffic jam or work in progress in a road network, temporary closure of an airport in an airline network and so on. In a biological network one or more nodes (genes, proteins, metabolites) are possibly removed from the network because of gene deletion, pharmacological treatment or protein degradation. For instance, in the case of a pharmacological treatment, it is possible to infer side effects of a drug by looking at the topological properties of nodes in a drug-treated network, meaning with that a network in which a drug-targeted node (protein) was removed [19]. Similarly we can simulate the consequences of gene deletions, which implies loss of coding genetic material and corresponding encoded proteins, thus resulting in the removal of one or more nodes from the network. The robustness notion is complementary to the interference one. It is computed evaluating the interference of all the nodes in the network with respect to a single target node. This allows identifying the node or the group of nodes that more than others affect the functionality of a selected node, and if its role is dependent on any particular node. In the next section we describe the interference and robustness computation methodology along with few explanatory examples. Following, we describe a case study, corroborated by data derived from an experimental setting of in vitro human leukocyte integrin activation, showing how node interference and robustness can predict network functionality and the effects of network modifications.

## Results and Discussion

### Nodes Centralities Interference: Definition

Due to its importance and wide diffusion for applications in several fields of science we focus on node interference for the betweenness centrality index [20],[21],[22],[23],[24],[16],[25],[26],[27],[28],[29],[30],[31],[32],[33],[34],[35],[36]. Following, the results are extended to other centrality indexes (see File S1). All definitions consider connected networks (i.e. networks where each node is reachable from all the others), which remain connected even after node removal. This hypothesis is in agreement with results in attack tolerance for scale-free networks [14].

**Figure 1:**
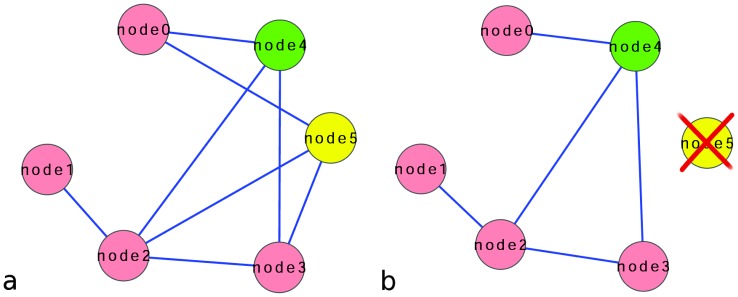
Betweenness interference. **a**. Node5 and node4 are in the shortest paths from node0 to the other nodes. **b**. Node5 have been removed. Node4 is now essential for connecting node0 to the rest of the network: it is the only node in the shortest paths connecting node0 to the other nodes: node4 betweenness increases.

Given a network 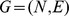 where 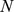 is the set of nodes and 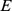 is the set of edges we consider the betweenness centrality and its relative value i.e. the value normalized by the sum of the betweenness of all the nodes (see Materials and Methods). This give the fraction of betweenness of each node with respect to the rest of the network. To introduce the notion of betweenness interference we consider the network in figure 1a. Node0 is connected to the rest of the network through nodes node4 and node5. If we remove node5 from the network, node4 become the only node connecting node0 to all the other nodes of the network (figure 1b), consequently its betweenness value will increase. This is a case of 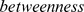 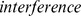 of node5 with respect to node4 since there is “interference” of node5 with respect to the betweenness value of node node4. Such interference, and the interference of node5 with respect to all the other nodes, is detected by removing node5 from the network and can be measured as follow: 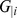 is the network obtained from 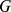 removing node 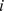 and all its edges from the network. The 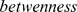 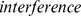 of a node 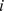 with respect to another node 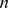 in the network 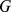 is:
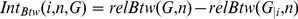
(1)


The measure shows which fraction of betweenness value a node loses or gains with respect to the rest of the network when the node 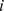 is removed. The definition is not symmetric and in general we have 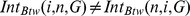. Notably, expressing interference values as percentage may facilitate understanding the meaning of the calculated data. The complete analysis of the network in the example is shown in table 1.

**Table 1:** Interference values of the network in figure 1, expressed as percentage.

Nodename	Betweenness(with node5)	Betweenness(node5 removed)	Interferencevalue
node0	4.167	0.000	4.167
node1	0.000	0.000	0.000
node2	54.167	50.000	4.167
node3	4.167	0.000	4.167
node4	18.750	50.000	−31.250
node5	18.750		

As expected node5, node4, and node2 have high betweenness value (first column). Node5 has negative interference with respect to node4. If it is removed from the network, node4 gains more than 30% of the total betweenness value (from 19.00 to 50.00). This is reflected by the negative sign of interference (−31.00): the presence of node5 is negative for node4 to play a central role in the network.

### Positive and Negative Interference

**Figure 2:**
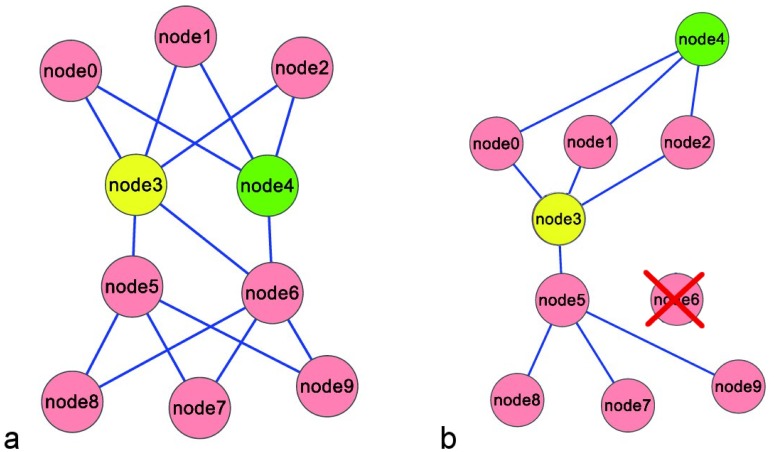
Positive and negative interference. **a** Node3 and node4 are the nodes connecting the top of the network with the bottom. **b** Node6 has been removed: node4 becomes a peripheral node, its betweenness decreases. The presence of node6 is important for node4 to play a central role (positive interference). At the same time, node3 and node5 become fundamental connections betweenn the top and the bottom. Their betweenness values increase. The presence of Node6 in the network on the left damages the “central role” of node3 and node5 (negative interference).

As in the example of figure 1, the 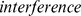 value of a node 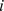 with respect to a node 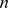 can be positive or negative. The example of the network in figure 2, explains the difference of the two notions of positive and negative interference.

#### Positive interference

If a node (n), upon removal from the network of a specific node (i), decreases its value for the considered centrality index, its interference value is positive. This means that this node (n), topologically speaking, takes advantage (is positively influenced) by the presence in the network of the node (i). Thus, “removal” of node (i) from the network, negatively affects the topological role of the node (n). This is called positive interference. For instance, consider Node4 in figure 2. It has high value of betweenness (15% of the total, see table 2), since it is important to connect the top of the network with the bottom. But this importance strictly depends on node6. Indeed, by removing node6, node4 results a peripheral node, as shown in figure 2b, and its betweenness consistently decreases (from 15% to 3.57% of the total. See table 2). This is a typical case of “positive interference”, since the high betweenness of node4 depends on the presence of node6: if node6 is part of the network node4 has higher betweenness value.

**Table 2:** Node6 and Node9 interference values of the network in figure 2, expressed as percentage.

Node	BetweennessNetwork a	BetweennessNetwork b	Node6Interference	Node9Interference
node0	0.98	3.97	−2.99	−0.407
node1	0.98	3.97	−2.99	–0.407
node2	0.98	3.97	−2.99	−0.407
node3	32.05	41.67	−9.61	–4.613
node4	15.00	3.57	11.43	−1.667
node5	15.00	42.86	−27.86	−3.333
node6	32.05			−4.554
node7	0.98	0.00	0.98	–0.685
node8	0.98	0.00	0.98	−0.685
node9	0.98	0.00	0.98	
		Globalinterference	60.80	16.758
		MaxInterference	27.857	4.613

The highest positive interference is with respect to node4. This node is more important if node6 is part of the network. The highest negative interference values are with respect to node5 and node3. These become part of the unique connection between the top and the bottom of the network when node6 is removed. The presence of node6 is negative for these nodes to have a “central” role.

#### Negative interference

If a node (n), upon removal from the network of a specific node (n), increases its value for the considered centrality index, its interference value is positive. This means that this node (n), topologically speaking, is disadvantaged (is negatively influenced) by the presence in the network of the node (n). Thus, “removal” of node (i) from the network, positively affects the topological role of node (n). This is called negative interference. For instance consider node3 in figure 2a. It is evident from the graphical representation that node3 plays a role similar to node4: they both connect the top of the network with the bottom, and they can be considered “competitors” in playing such a role. When removing node6, (fig. 2b), node3 remains the only node connecting the top with the bottom and its betweenness value increases (from 32.05% to 41.67% of the total. See table 2). This is a case of negative interference of node6 with respect to node3, since the presence of node6 negatively affects the central role of node3 in the network: node3 is more central if node6 is not part of the network thus node6 negatively interferes with node3 (betweenness values are reported in table 2).

A further step for a complete analysis of interference is to quantify the interference of a single node with respect to the global network architecture. In this case the goal is to quantify the influence of a node 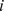 on the global topology of the network. Indeed, a node can have low interference value with respect to few nodes but can interfere significantly with the majority of the nodes in the network. In this case the node can be more relevant to the overall network topology (and, possibly, functionality) than to the topology of few nodes. In order to quantify the interference with respect to the entire network we can use the 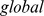 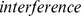 value defined as the sum of all the interference values of a node and the 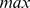 of the interference values (see File S1). If the max of the interference is high, it means that at least one node is consistently affected by node 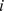. If the global interference value is high, it can be supposed that the node interferes with high values with respect to the a great number of nodes in the network. Consider Node9 in the network of figure 2. Node9 is a peripheral node and this is reflected by the low values of global interference and max interference, if compared for example with the same values of node6 (respectively 16.758 vs 60.800 and 4.613 vs 27.857 see table 2). Indeed the removal of node9 does not significantly affects the global structure of the network.

### Nodes Centralities Robustness. Who is Affecting a Node?

We now describe node robustness, the reverse problem of interference. As above, we focus on betweenness. Here the emphasis is not on the effects of an individual node removal on the network, but on how other nodes can affect the functionality of a specific node. This corresponds to ask whether a node is resilient to modification of the network. To answer to this question, we introduce the notions of node 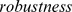, 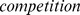 and 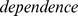. The betweenness robustness of node 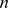 is obtained by computing all the interference values from the other nodes with respect to node 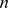 and is defined as
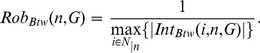
(2)


It depends on the maximum interference value affecting the betweenness value of the node. If it is low, the node can be easily “attacked” by removing particular nodes. If it is high, the node is “robust”, i.e. there is no node removal that can affect its betweenness value and consequently its functionality. Notably, we consider the absolute value of interference. Similarly to 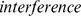, positive and negative 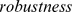 can be defined (see File S2) but it is more intuitive to consider their reciprocal values respectively 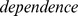 and 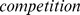 values. The 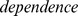 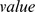 of a node 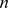 is
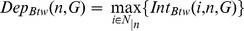
(3)where 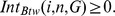 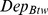 is the maximum over the positive interference values. If high it means that the node is “central” because of the presence of at least another node in the network: if that node is removed then node 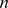 loses a consistent part of its central role (its betweenness value decreases). It is the case of node4 in the network of figure 2 where it has a central role depending on node6. When node6 is removed node4 becomes a peripheral node: it strongly depend on node6 (see fig. 2b). If the dependence value of a node 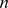 is low, its central role is not dependent on other nodes and there is no node removal that can consistently affects its relevance in the network. Similarly we define the 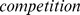 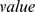 of a node 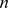 as
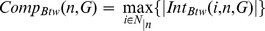
(4)where 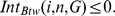 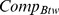 is the maximum over the negative interference values. High competition value means that the central role of node 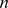 can be “improved” removing a particular node from the network (node 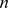 betweenness increases). In this sense the two nodes, node 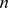 and the removed one are “competitors” in the network. It is the case of node3 and node4 of the network in figure 2. Removing node3, node4 becomes the unique node connecting the top and the bottom of the network, and conversely removing node4: node3 and node4 are “competitors” in the role of connecting the two parts of the network. If the competition value is low, the central position of the node cannot be improved removing a particular node from the network. To improve the significance of the betweenness variation expressed by the robustness analysis, the competition and dependence values can be also related to the betwenness of the node in the starting network (the network with no node deletion, see File S2). Total robustness, dependence and competition can be also used as global parameters in order to characterize the entire network (see File S2).

#### Interpretation of robustness analysis

Consider again the network in figure 2a. Its betweenness values are reported in table 2. Node3 and node6 have the highest value of betweenness (32.05), node4 and node5 present the third highest value (15). The robustness analysis of node3 and node4, reported in table 3, allows understanding if and how much their high betweenness values depend on other nodes. Node4 has a dependence value of 11.429, higher than node3 which is 9.995. The reason of this difference is that even if they have both the role of connecting the top and the bottom of the network, if we remove node6, node4 becomes a peripheral node, while node3 mantains its connecting role because it is connected also to node5. Thus the dependence value of node4 is higher and only mediated by node6. The highest dependence value of node3 is due to node5: if we delete node5, node3 still remains a connecting node, but its betweenness become the same of node4, since they connect the same nodes through the same paths, those passing through node6. Also the competition value of both nodes is very informative. The highest value of node3 depends on deletion of node4 (21.623) and the highest value of node4 depends on node3 (27.857). In this sense they are really “competitors” in the network.

**Table 3:** The robustness analysis of node3 and node4.

node name	interf. w.r.t. node3	node name	interf. w.r.t. node4
node5	9.995	node6	11.429
node0	4.554	node0	3.333
node1	4.554	node1	3.333
node2	4.554	node2	3.333
node7	−4.613	node7	−1.667
node8	−4.613	node8	−1.667
node9	−4.613	node9	−1.667
node6	−9.613	node5	−7.059
node4	−21.623	node3	−27.857
node3		node4	
robustness	0.046	robustness	0.036
dependence	9.995	dependence	11.429
competition	21.623	competition	27.857

Interference values are expressed as percentage. The robustness, competition and dependence values are inferred from the interference values of all the nodes with respect to node3 (left) and node4 (right). Node4 is highly dependent on node6 (11.429) more than node3 on node5 (9.995). Note the high competition values of node3 due to node4 and the high competition values of node4 due to node3, denoting their high reciprocal influence.

But this also means that, if one of the two nodes is missing, the role of the remaining node can be replaced by the other, and in this sense we can say that they have similar roles. The robustness value also confirm that node3 is nore resilient to node deletion than node4 (0.046 vs 0.036).

The notion of interference and robustness can be applied to other centrality measures (see File S1 for extended definitions). An example of application of interference to a kino-phosphatome network [11], a network of human kinases and phosphatases can be found in the File S3 and File S8). The notion of Interference and Robustness can also be applied to directed networks. Even if some centralities definition cannot be applied to directed networks, we recently released CentiScaPe 2.1 [11] where several centralities parameters definition are modified to be used in directed networks. Such definitions (see File S1) can be used for a directed version of the interference and robustness notion, in order to be used in directed biological networks as for example signal transduction or metabolic networks. In the next section we discuss the application of interference and robustness to a network of signaling proteins regulating the leukocyte integrin activation process.

### Interference in a Protein-protein Interaction Signalling Network Regulating the Immune Response

**Figure 3:**
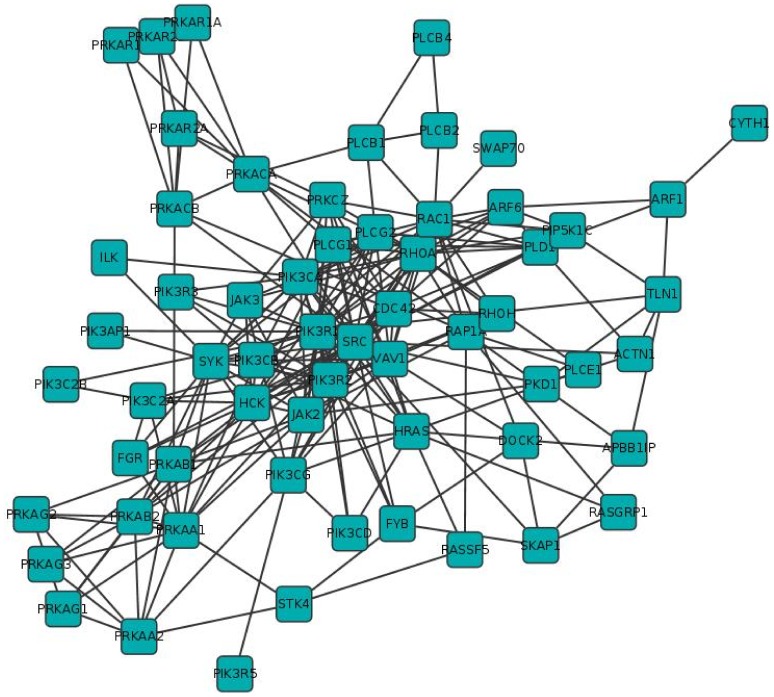
Integrin network. The network of the 61 integrins and 241 binary interactions, involved in the process of adhesion regulation.

**Figure 4:**
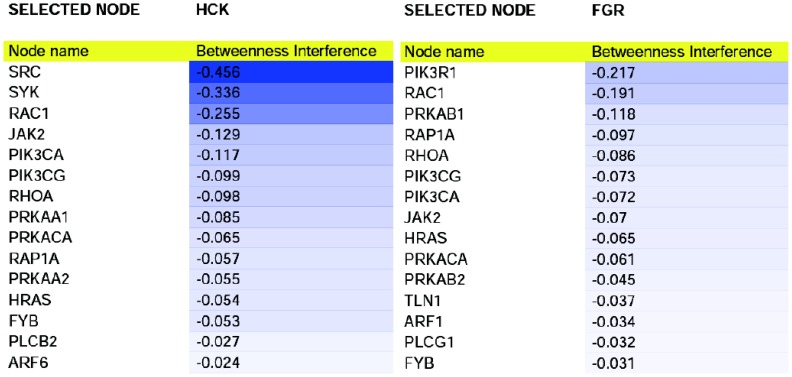
Interference of HCK and FGR. First fifteen negative interference values of HCK and FGR, expressed as percentage.

**Figure 5:**
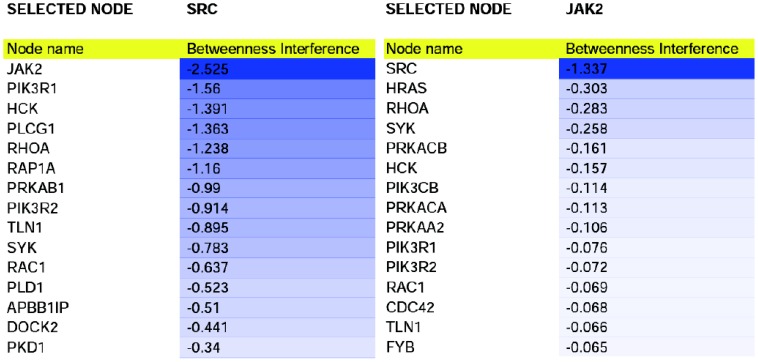
Interference of SRC and JAK2. First fifteen negative interference values of SRC and JAK2, expressed as percentage. Note the reciprocal high interference of SRC and JAK2.

**Figure 6:**
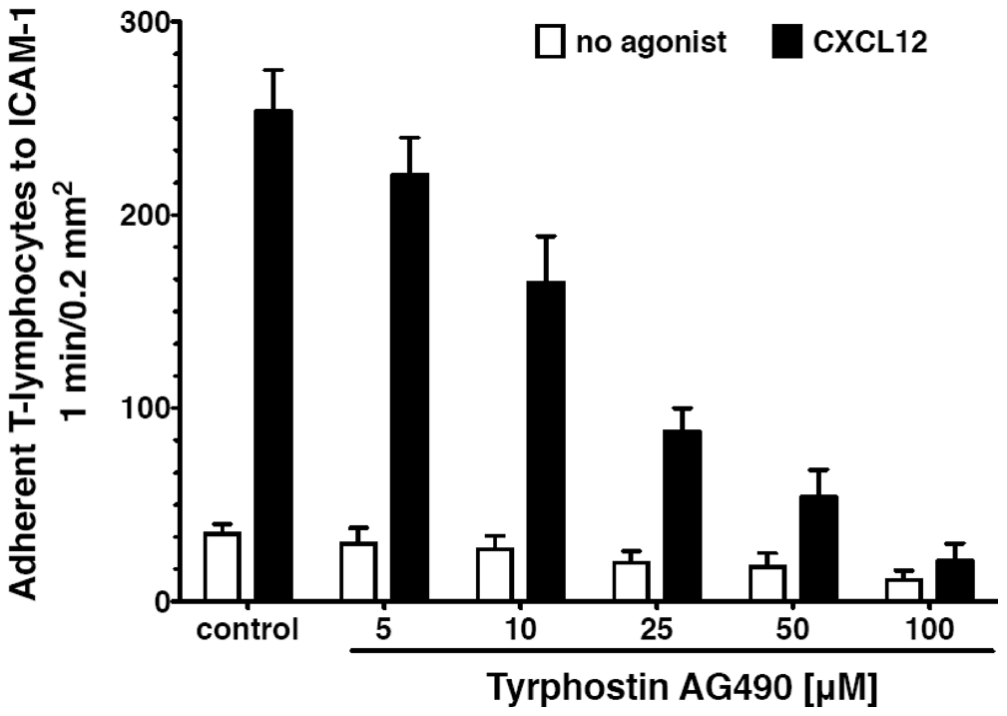
Effects of Jak2 inhibition. Lymphocytes were pre-treated with the indicated concentrations of tyrphostin AG490 at 37°C for 15 min and stimulated with 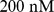 CXCL12 for 1 min. Plots are averaged values of adherent 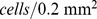 with standard deviation N = 5.

The modality of network analysis described in previous sections can be applied to biological networks analysis. To easily apply the interference and robustness notions to real networks, we developed the Interference plugin for the Cytoscape platform, based on the existing CentiScaPe plugin [11] for node network centrality calculation. We analyzed an intracellular protein-protein interaction network regulating the immune response. Particularly, we analyzed a sub-network of the human protein interactome involved in adhesion modulation during the process of leukocyte recruitment. Leukocyte recruitment is the “primu movens” of every immune response and consists of a complex sequence of cellular events leading to leukocyte accumulation into sites of inflammation [37]). Each step of this homeostatic mechanism is finely regulated by combinatorial molecular mechanisms. A central event is the regulation of a family of activable adhesion surface receptors called integrins. Integrin activation is mandatory to the completion of leukocyte recruitment and is regulated by chemoattractants, which, in turn, trigger an intricate network of signaling proteins devoted to integrin function modulation. Till now, at least 61 intracellular signaling molecules have been shown to be involved in positive or negative modulation of leukocyte adhesion [38], each one potentially interacting with a number of upstream regulators and downstream effectors. In this context, it is of pharmacological interest to be able to identify which proteins are most suitable for potential target anti-inflammatory therapies. Here the interference analysis may allow a qualitative prediction of such potential target proteins identifying which of them are candidates to be mostly affected by the inhibition of one or more nodes. Since the topological structure of biological networks reflects functionality [39]; [40], we wished to verify, in the context of signaling events regulating integrin activation, whether network centrality interference might unveil the prominent functional relevance of specific signaling proteins. We followed the same logic normally applied in experimental biology to identify a cause-effect relationship. Accordingly, a cell is treated with a pharmacological inhibitor specific for a certain signaling molecule and the effect on the regulated cell phenomenon is quantified. In our case, a virtual pharmacological treatment is achieved by removing a node and calculating the interference on the centrality of the remaining nodes. To achieve this goal, we reconstructed the interactomic network generated by the 61 known molecules (see fig. 3 and File S4) and calculated the specific interference of selected proteins on centrality indexes of all other proteins involved in adhesion regulation. The network consisted of 241 unique binary interactions (see Materials and Methods). As proof of concept, we focused our analysis on the betweenness interference of 3 kinases: SRC, HCK and FGR. We focused on these related kinases, as they are negative regulators of integrin affinity maturation and specific pharmacological inhibitors are widely available and, thus, their analysis may provide more meaningful, testable, data. The three kinases have either negative as well as positive interference on the network (see File S5). The analysis highlighted that SRC, HCK and FGR have the highest negative interference on a group of 15 proteins including JAK2, PIK3R1, PIK3R2, PIK3CG, HCK, PLCG1, RHOA, RAP1A, RAC1, PRKAB1, TLN1, SYK, PLD1, SRC and HRAS (see fig. 4 and 5). Thus, the presence of SRC, FGR and HCK in the network modulating integrin activation negatively affects the topological role, quantified as betweenness, of these proteins. Importantly, this result is perfectly in keeping with published experimental data. Indeed, RHOA, RAC1, PLD1, RAP1A, HRAS, TLN1, PIK3R1, PIK3R2 and PIK3CG, are well-known positive regulators of integrin triggering [38]; [41], which is the crucial step in leukocyte arrest in vivo [42]; [43]. In this context, the analysis is fully confirmed by published experimental data, showing that FGR and HCK play a negative role in integrin affinity triggering [44]. Thus, the negative role of FGR and HCK, detected at topological level, fully reflects their biological activity. Notably, the negative interference on PLCG1 is also coherent with published data, since PLCG1 may generate second messengers leading to RAP1A activation, whose positive role in integrin triggering has been widely demonstrated [45]. Intriguingly, SRC and FGR interference on HRAS are opposite. However, HRAS role in integrin modulation is more complex, showing both positive and negative activities [46]. It is also of interest the reciprocal negative interference of SRC and HCK whereas, in contrast, FGR displays a positive interference on HCK and SRC, suggesting hierarchy and possible competition for similar effectors between these tyrosine kinases. This is not completely surprising since SRC, HCK and FGR, are highly structurally and functionally related kinases. Notably, the analysis also highlighted that SRC, HCK and FGR have positive interference on a group of proteins including, among the others, PRKACB, PRKAB2, SKAP1, ARF6, HRAS, SRC, SYK, HCK, VAV1 and CDC42. Here, CDC42 is of specific interest. Indeed, it was recently demonstrated that CDC42 has a negative regulatory role on rho-mediated integrin affinity triggering [41], similarly to the role of FGR and HCK. Thus, the fact that the presence of HCK in the network leading to integrin activation positively affects the topological role of CDC42, quantified as betweenness, further confirms the correspondence between topological interference and biological activity. Interestingly, JAK2 resulted the most sensitive to SRC negative role, and rather sensitive to FGR and HCK negative roles. This could suggest that JAK2 is an important positive player in the overall mechanism of integrin activation. Importantly, the involvement of JAK-related tyrosine kinases in leukocyte trafficking was previously suggested, although in mouse and with rather indirect evidence [47]. Thus, to corroborate the prediction of an involvement of JAK2 in the regulation of integrin activation suggested by the interference computation, we set out to experimentally verify whether the computed JAK2 interference does correspond to the experimental biological outcome. To this end, we measured the effect of tyrphostin AG490, a well-known, specific, JAK PTKs inhibitor on adhesion triggering by chemoattractants in human primary T-lymphocytes (see Materials and Methods and [41]). As shown in fig. 6, AG490 prevented in a dose-dependent manner rapid adhesion to ICAM-1 of human primary T-lymphocytes, thus confirming the involvement of JAK2 in integrin triggering and corroborating the prediction of the interference analysis. Moreover, the role of JAK2 is confirmed also by the JAK-dependent negative interference on the other molecules in the network (see fig. 5 and File S6). The highest interference value of JAK2 is with respect to SRC. Thus, SRC and JAK2, that have been experimentally detected respectively as integrin inhibitor and activator, have reciprocal high interference. Importantly, the key role of the proteins in the network is also corroborated by their robustness analysis reported in Table S1. Here, we computed all the interference values affecting JAK2 and SRC. Among all proteins, JAK2 is mostly affected by SRC with a negative interference value of −2.525. Then, JAK2 is the second highest negative interference with respect to SRC with −1.337 (the first is PIK3R1 with −1.648). Thus, there is not only a reciprocal influence between the two proteins, but it is the highest between all the proteins of the network. Note that SRC and JAK2 are direct interactors. They have respectively 27 and 15 direct interactors for a total of 31 different proteins (11 are common interactors). The interference analysis allowed identifying SRC and JAK2 as the two most important interactors in a set of 31 nodes. High dependence of SRC on SKAP1 and ACTN1 (respectively 1.277 and 1.175) and competition value of SRC due to PIK3R1 should be further experimentally investigated. JAK2 is also dependent on RAP1A but with a less significant value (0.792). The interference analysis indicates SRC and JAK2 as preferential targets for further experiments. Also the average and max interference values are interesting. In Table S2 are reported all the average and max interference values. Among all proteins PIK3CD, PIK3R3, RHOH, PRKAG3, PRKAG2, PRKAG1 have both the max and average values in the last 20 scores. This possibly suggests that they may have a marginal role in the integrin activation as also showed by previous studies [37], [38]. Not all data of the interference analysis could be linearly interpreted, such as the role of SYK or PRKACB and PRKAB2, likely due to complexity of the network and lacking of experimental data.

## Conclusions

In this study we introduced the new centralities based notions of interference and robustness. The interference notion allows identifying the context of major influence of a particular node, removing it from the network and evaluating the effects of the removal as a variation of centrality values. If applied to a protein-protein interaction network, this is similar to perform a virtual knock-out experiment on the network allowing topologically predicting side effects of protein inhibition. Conversely, the dual notion of interference is the robustness notion that allows evaluating how much a node is influenced by other nodes removal. A robust node is a node that is minimally affected by the removal of other nodes from the network. The effects of proteins inhibition in the integrin activation process have been predicted by the interference analysis performed in the study suggesting new perspectives in biological network analysis such as the possibility of predicting potential side effects of drugs or the characterization of the consequences of genes deletion, duplication or of proteins degradation in biological process. Indeed, given the proper protein-protein network, the output of the interference and robustness analysis possibly unveils non trivial functional interactions between proteins (as in the case of SRC and JAK2) contributing to a qualitative comprehension of the biological process and thus driving further experiments. The method can be applied to both undirected and directed networks not only in biological contexts, and is mainly limited by the overall quality of the analyzed networks. The method gives also the opportunity to explore the robustness of biological networks and the relation betweenn strongly connected components or cliques and the variation of network centralities. In clique where the nodes are all connected or in strongly connected components we expect that removal of nodes has a low effect on the centralities of other nodes. So nodes beeing part of a strongly connected network should have high robustness and low interference. In biological networks (that are robust) when nodes are randomly removed the effects are low but become relevant when important nodes are removed [14]. A future works should consider to use the interference values to identify nodes that are part of a strongly connected components or that connects different strongly connected components. Similarly, the effects of removal nodes in network motifs should be furtherly investigated. Network motifs are variable for structure and function, so they need dedicated studies to be related to the variation on the centralities values when removing nodes. The removal of one node in a motif can completely destroy a motif (and its biological role) or can have no impact depending on its structure and on the removed node. Further studies are necessary to identify which network motifs are mostly affected by node removal and to relate them to the interference notion.

## Materials and Methods

### Betweenness Definition

We consider a network as a graph 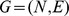 where 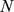 is the set of nodes and 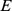 is the set of edges. Betweenness of node 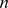 is defined as
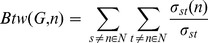
(5)where 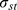 is the number of shortest paths between 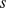 and 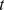 and 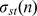 is the number of shortest paths between 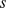 and 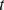 passing through the node 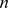. We consider the relative value of betweenness by normalizing it as
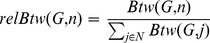
(6)in order to have the fraction of betweenness of each node with respect to the rest of the network.

### Software: the Interference1.0 Cytoscape Plug-in

To calculate the interference values the Interference.1.0 Cytoscape plugin have been developed and released. The software is based on the last version of our CentiScaPe plug-in [11], developed to compute several nodes centralities. The Interference.1.0 computes the interference for Betweenness, Stress, Closeness, Eccentricity, Radiality and Centroid Values. The software calculate also the min, max and average values of interference. Results are displayed as Cytoscape attributes or as an heatmap that can be exported in pdf format. The Interference values can be also computed for set of nodes (i.e. removing more than one node at the same time. See File S7 for interference definition extended to subset of nodes). The plugin is available at: http://www.cbmc.it/%7Escardonig/interference/Interference.php or via the Cytoscape website. Computational complexity of the algorithm for centralities values is 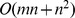 except for betweenness where the algorithm used is 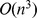 (n is the number of nodes, m the number of edges). This can results in a long computation time for large networks, depending on the characteristics of the computer used. The interference computation requires two computation for each centrality doubling the computation time. Moreover, the robustness computation requires to calculate the interference values for each node, resulting in a computational complexity of 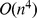 for betwenness interference and of 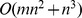 for the other centralities further increasing the computation time.

### Integrin Network

To apply the interference analysis we reconstructed the protein-protein interaction network generated by the 61 known molecules involved in the positive or negative modulation of leukocyte adhesion [38]. The network of the known interaction betweenn these proteins have been obtained crossing the information from six different databases (HPRD, BIND, DIP, IntAct, MINT, BioGRID) and consists of 241 unique binary interactions (File S7). To limit the presence of false positive, the interactions are considered only if they are validated by two or more databases, and by two to six proteomics methods. Otherwise, if an interaction is present in only one database it is considered as a potential false positive and have not been added to the network.

### Ethics Statement

Samples were collected under a protocol approved by Ethics Committee of the Azienda Ospedaliera Universitaria Integrata of Verona, Italy (Comitato Etico per la Sperimentazione AOUI) and data were analyzed anonymously. In accordance with the Declaration of Helsinki, all donors provided written informed consent for the collection and use of their blood samples for research purposes.

### Lab Experiment: JAK2 Inhibition

To test the involvment of JAK2 in integrin triggering we measured the effect of tyrphostin AG490, a well-known JAK-specific inhibitor on adhesion triggering by chemoattractants in human primary T-lymphocytes. Human primary T-lymphocytes were isolated from healthy donors and rapid static adhesion assays on ICAM-1 have been performed as reported in [41]. Lymphocytes were pre-treated with the indicated concentrations of tyrphostin AG490 at 37°C for 15 min and stimulated with 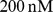 CXCL12 for 1 min.

## Supporting Information

Table S1 **Robustness analysis of JAK2 and SRC in the integrin activation network.**
(XLS)

Table S2 **Average and Max Interference values for the integrin activation network.**
(PDF)

File S1 **General definition of Interference.** Definition of max, global, mean Interference. Centralities definition for directed and undirected graph.
(PDF)

File S2 **Definition of centralities Robustness, Dependence and Competition value.**
(PDF)

File S3 **Example of Interference analysis of the Human Kino-Phosphatome network.**
(PDF)

File S4 **Integrin network in.sif format.**
(SIF)

File S5 **Betweenness Interference values for SRC FGR and HCK in the integrin network.**
(PDF)

File S6 **Betweenness Interference values for JAK2 in the integrin network.**
(PDF)

File S7 **Interference definition extended to subset of nodes.**
(PDF)

File S8 **The Kino-Phosphatome network, sif format.**
(SIF)
