# Supplementary material for: Node Interference and Robustness: Performing Virtual Knock-Out Experiments on Biological Networks: The Case of Leukocyte Integrin Activation Network
Source: PLoS One. 2014 Feb 20;9(2):e88938. doi: 10.1371/journal.pone.0088938 (PMC3930642; doi:10.1371/journal.pone.0088938)
Supplement: Table S2 — Average and Max Interference values for the integrin activation network. (PDF) [file pone.0088938.s002.pdf]

1 Suppl. Table S2: Average and max interference values

Average interference		node name	Interference max value
SRC	-0.265	PRKACA	3.793
PIK3R1	-0.141	PRKACB	2.829
PRKACA	-0.083	SRC	2.525
HRAS	-0.081	CYTH1	2.311
PRKAB1	-0.064	PIK3R5	2.240
RHOA	-0.062	HRAS	2.209
PRKAA1	-0.060	SWAP70	1.990
PRKACB	-0.055	RAP1A	1.968
SYK	-0.053	PLCB2	1.912
RAP1A	-0.052	PIK3R1	1.793
JAK2	-0.048	PLCB1	1.631
PIK3CA	-0.042	PIK3C2B	1.585
PLD1	-0.035	PLD1	1.574
ARF6	-0.034	PLCB4	1.544
PLCB1	-0.033	ARF6	1.449
VAV1	-0.028	TLN1	1.411
PIK3R2	-0.027	PRKAB1	1.384
HCK	-0.025	JAK2	1.337
PRKAB2	-0.024	SKAP1	1.277
PRKCZ	-0.024	PRKAR2A	1.269
FYB	-0.020	PIK3AP1	1.199
TLN1	-0.020	ILK	1.188
PLCG2	-0.018	ACTN1	1.175
PLCG1	-0.017	PIK3CA	1.170
PRKAA2	-0.015	PRKAR1A	1.145
CDC42	-0.013	PRKAR1B	1.145
PLCB2	-0.012	PRKAA1	1.123
PIK3CB	-0.012	PLCG2	1.055

Table 1: Interference average and max values. PIK3CG, RAC1 and ARF1 deletion results in a disconnetted network. The interference and robustness cannot be calculated for these proteins.

Average interference		node name	Interference max value
PKD1	-0.003	APBB1IP	0.818
ACTN1	-0.002	FGR	0.781
PIK3CD	-0.001	PIK3R2	0.754
RASGRP1	-0.002	PKD1	0.777
PIK3C2B	-0.001	DOCK2	0.750
PLCE	0.000	PRKAG3	0.731
FGR	0.000	PIK3C2A	0.728
SKAP1	-0.010	RASSF5	1.043
PIK3C2A	-0.009	PRKAA2	1.039
STK4	-0.008	PRKAR2B	1.038
APBB1IP	-0.007	RASGRP1	1.030
RASSF5	-0.005	SYK	0.981
PIP5K1C	-0.004	PRKCZ	0.916
PRKAR2A	-0.004	PLCE1	0.871
DOCK2	-0.004	STK4	0.853
JAK3	-0.003	RHOA	0.833
PIK3R3	0.000	PRKAG1	0.722
CYTH1	0.000	PRKAG2	0.722
PIK3AP1	0.000	PRKAB2	0.701
ILK	0.000	VAV1	0.692
RHOH	0.000	PIK3R3	0.672
PRKAR1B	0.000	FYB	0.664
PRKAR1A	0.000	PIK3CD	0.646
PIK3R5	0.000	PLCG1	0.607
SWAP70	0.000	PIP5K1C	0.591
PRKAG3	0.000	HCK	0.456
PRKAG2	0.000	PIK3CB	0.456
PRKAG1	0.000	RHOH	0.427
PLCB4	0.000	JAK3	0.412
PRKAR2B	0.000	CDC42	0.201

Table 2: Interference average and max values. PIK3CG, RAC1 and ARF1 deletion results in a disconnetted network. The interference and robustness cannot be calculated for these proteins.
