# Supplementary material for: Node Interference and Robustness: Performing Virtual Knock-Out Experiments on Biological Networks: The Case of Leukocyte Integrin Activation Network
Source: PLoS One. 2014 Feb 20;9(2):e88938. doi: 10.1371/journal.pone.0088938 (PMC3930642; doi:10.1371/journal.pone.0088938)
Supplement: File S1 — General definition of Interference. Definition of max, global, mean Interference. Centralities definition for directed and undirected graph. (PDF) [file pone.0088938.s003.pdf]

Suppl. File S1

1 Centrality interference definitions

Interference centralities definitions are generalized to other centralities as Stress, Radiality, Closeness, Eccentricity and Centroid value as follows. The general definition can be also applied to directed networks, using the proper centralities definition. Given a centrality C the relative interference (or simply interference) of the node i with respect to node n in the network G is:

$$Int_C(i,n,G) = \frac{C(G,n)}{\sum_{j\in N} C(G,j)} - \frac{C(G_{|i},n)}{\sum_{j\in N} C(G_{|i},j)}$$

Note The relative interference definition cannot be applied to centroid value since it can have also negative values. To apply it to the centroid value all the values are shifted by the minimum of the centroid values + 1. This results in a vector of only positive values.
In order to quantify the interference with respect to the entire network we introduce the max interference value and the global interference value. They can easily derived from the previous definitions. The interference max value of node i with respect to the network G is defined as follow:

$$maxInt_C(i,G) = \max_{n\in N_{|i}} \left\{ \left| \frac{C(G,n)}{\sum_{j\in N} C(G,j)} - \frac{C(G_{|i},n)}{\sum_{j\in N} C(G_{|i},j)} \right| \right\}$$

Then we define global interference value of node i

$$Int_C(i,G) = \sum_{n\in N_{|i}} \left(\frac{C(G,n)}{\sum_{j\in N} C(G,j)} - \frac{C(G_{|i},n)}{\sum_{j\in N} C(G_{|i},j)} \right)$$

and mean interference value

$$meanInt_C(i,G) = \left(\sum_{n\in N_{|i}} \left(\frac{C(G,n)}{\sum_{j\in N} C(G,j)} - \frac{C(G_{|i},n)}{\sum_{j\in N} C(G_{|i},j)} \right) \right) \frac{1}{|N|-1}$$

where $|N|$ is the number of nodes of the network.

2 Centralities for undirected graphs

Here are all the centralities definitions for undirected graphs

2.1 Preliminary definitions

Let $G = (N, E)$ an undirected graph, with $n = |N|$ vertexes. $dist(v, w)$ is the shortest path between v and w. σ_{st} is the number of shortest paths between s and t and $\sigma_{st}(v)$ is the number of shortest paths between s and t passing through the vertex v. Notably:

2.2 Eccentricity ($C_{ecc}(v)$)

$$C_{ecc}(v) := \frac{1}{max\{dist(v,w) : w \in N\}}$$

2.3 Closeness ($C_{clo}(v)$)

$$C_{clo}(v) := \frac{1}{\sum_{w \in N} dist(v,w)}$$

2.4 Radiality ($C_{rad}(v)$)

$$C_{rad}(v) := \frac{\sum_{w \in N} (\Delta_G + 1 - dist(v,w))}{n-1}$$

where Δ_G is the diameter of the network (max of the shortest paths).

2.5 Centroid value ($C_{cen}(v)$)

$$C_{cen}(v) := min\{f(v,w) : w \in N\{v\}\}$$

Where $f(v,w) := \gamma_v(w) - \gamma_w(v)$, and $\gamma_v(w)$ is the number of vertex closer to v than to w.

2.6 Stress ($C_{str}(v)$)

$$C_{str}(v) := \sum_{s \neq v \in N} \sum_{t \neq v \in N} \sigma_{st}(v)$$

2.7 S.-P. Betweenness ($C_{spb}(v)$)

$$C_{spb}(v) := \sum_{s \neq v \in N} \sum_{t \neq v \in N} \delta_{st}(v)$$

where

$$\delta_{st}(v) := \frac{\sigma_{st}(v)}{\sigma_{st}}$$

3 Centralities for directed graphs

Here are all the centralities definitions for directed graphs

3.1 Preliminary definitions

Let $G = (V, E)$ a directed graph, with $n = |V|$ vertexes. $deg(v)$, $deg(v)$, $deg+(v)$ indicate, respectively, the degree, the in-degree and the out-degree of the vertex. $dist(v, w)$ is the shortest path between v and w. σ_{st} is the number of shortest paths between s and t and $\sigma_{st}(v)$ is the number of shortest paths between s and t passing through the vertex v.

A directed network is a network where the edge has a direction. This means that given two nodes A and B, some times there are no paths betweens them, or there is a path from node A and B but not from node B to node A. This is very typical of signaling or metabolic networks, where informational or energy/mass flow follows a specific direction determined by the thermodynamics of biochemical reactions. Some of the centralities definitions cannot be used in directed networks, so they have been redefined in order to consider direction of the edges.

3.2 Eccentricity

The undirected definition is

$$C_{ecc}(v) := \frac{1}{max\{dist(v, w)\ :\ w\ \in\ V\}}$$

Eccentricity is calculated by computing all the shortest path between v and all the other nodes in the graph. Then the longest path is chosen and the eccentricity computed. For directed graphs the formula has a new definition; this is necessary because a node maybe never be reached by another node in the network. In this case the distance between the two nodes, that aren't connected, is set to infinity, giving a value of eccentricity equal to zero. Zero is the lowest value for eccentricity. The highest value is one, when the longest shortest path between v and all his neighbors is one.

This is the definition used in the new algorithm:

$$dist(v, w) := \infty\, if\ v\ doesn't\ reach\ w$$

3.3 Closeness

Closeness is quite similar to eccentricity, because both work on distances between nodes, but it is used to find the minimal sum of all the distances in a network. With eccentricity we want to minimize the longest path, instead with closeness we want to find the node that minimizes the distance to any other node in the graph. So nodes with high closeness values has low distances to their neighbors. The definition of closeness:

$$C_{clo}(v) := \sum_{w\,\in\,V} \frac{1}{dist(v, w)}$$

with:

$$dist(v, w) := \infty\, if\ v\ doesn't\ reach\ w$$

In directed networks, like in eccentricity centrality, we assume that a node that can't be reached has a distance from the root of infinity. This assumption permits to give a contribute to closeness sum that is near zero. This is important in biological networks because closeness can be viewed as the probability of a node to be more relevant for certain nodes and useless for the other. So a nodes with a high closeness, compared to the average closeness in the network, could be central in a process of regulation but irrelevant for a some few nodes. A network with a high average closeness is probably organized in functional modules, or clusters. At the opposite side a network with a low average closeness is probably an open cluster with proteins involved in different regulatory processes.

3.4 Radiality

Radiality, like closeness, is distance based and is useful to understand if a node is integrated into the network. This means that the closer the node is to other nodes then it is better integrated into the graph. Like closeness, high values of radiality suggest that the node can easily reach other nodes. This is the definition:

$$C_{rad}(v) := \frac{1}{\sum \Delta_G - \frac{1}{dist(v,w)}}$$

with:

$$dist(v, w) := \infty\, if\ v\ doesn't\ reach\ w$$

Radiality uses Δ_G that is the diameter of the graph that is the longest shortest path found in the network.

3.5 Stress

Stress centrality is based on shortest paths. Eccentricity, Radiality and Closeness are based on distances between node; this difference permits to evaluate those three centralities togheter and stress values are interpred using also Betweenness centrality, that is shortest path based. Stress gives us information about the number of shortest path passing through a node and tells us how much work has to do a vertex in a graph.

Shortest paths starting and ending in v are excluded because stress measure the paths that pass through a node. This assumption is used also for the Betweenness centrality. The value $\delta_{st}(v)$ is the number of paths that pass through a node starting from node s and ending in node t:

$$C_{str}(v) := \sum_{s \neq v \in V} \sum_{t \neq v \in V} \delta_{st}(v)$$

3.6 Betweenness

Betweenness like Stress is based on the number of shortest paths. This centrality is computed by counting the numbers of shortest paths starting from a node and ending in an other node, v_1 e v_2 that pass through a third node, n. The computing of betweenness is done by couples of nodes because two nodes are chosen and then the number of paths between them is counted. Then the value is compared to the number of this path that pass through a certain node, and then to another node for all the nodes in the network obtaining a partial betweenness. Then another couple is chosen and the computation restart. At the end we have to sum all partial betweenness for each node.

This centrality is interesting because it gives us not only the amount of work for every node, but also it tells us if a node is essential to maintain the connection in a graph.

$$C_{bet}(v) := \sum_{s \neq v \in V} \sum_{t \neq v \in V} \delta_{st}(v)$$

where:

$$\delta_{st}(v) := \frac{\sigma_{st}(v)}{\sigma_{st}}$$
