# Supplementary material for: Node Interference and Robustness: Performing Virtual Knock-Out Experiments on Biological Networks: The Case of Leukocyte Integrin Activation Network
Source: PLoS One. 2014 Feb 20;9(2):e88938. doi: 10.1371/journal.pone.0088938 (PMC3930642; doi:10.1371/journal.pone.0088938)
Supplement: File S2 — Definition of centralities Robustness, Dependence and Competition value. (PDF) [file pone.0088938.s004.pdf]

# 1 Suppl. File S2: centralities robustness, dependence and competition value

Given a network $G = (N, E)$ a centrality measure $C$ and a node $n \in N$, we define the centrality robustness of node $n$ as follow:

$$Rob_C(n, G) = \frac{1}{\max_{i \in N_{|n}} \{|Int_C(i, n, G)|\}}$$

Notice that we consider absolute value of interference. To consider only the positive interference we define positive robustness value as:

$$PosRob_C(n, G) = \frac{1}{\max_{i \in N_{|n}} \{Int_C(i, n, G)\}}$$

where

$$Int_C(i, n, G) \geq 0$$

To consider only the negative interference we define negative robustness as

$$NegRob_C(n, G) = \frac{1}{\max_{i \in N_{|n}} \{|Int_C(i, n, G)|\}}$$

where

$$Int_C(i, n, G) \leq 0$$

In some cases it is more intuitive to use the reciprocal of negative and positive robustness. We define the reciprocal of positive robustness as the *dependence value*:

$$Dep_C(n, G) = \max_{i \in N_{|n}} \{Int_C(i, n, G)\}$$

where

$$Int_C(i, n, G) \geq 0$$

We define the *competition value* as the reciprocal of negative robustness:

$$Comp_C(n, G) = \max_{i \in N_{|n}} \{|Int_C(i, n, G)|\}$$

where

$$Int_C(i, n, G) \leq 0$$

*relative robustness*, *relative dependence* and *relative competition* are defined as the fraction of the variation of the centrality value with respect to the starting centrality value. Given the centrality $C$ and a node $n$ the relative centrality value in the network $G$ is defined as:

$$relC(n, G) = \frac{C(n, G)}{\sum_{i \in N} Int_C(i, G)}$$

The relative robustness is

$$relRob_C(n,G) = \frac{relC(n,G)}{\max_{i\in N_{|n}}\{|Int_C(i,n,G)|\}}$$

Similarly for dependence value:

$$relDep_C(n,G) = \frac{Dep_C(n,G)}{relC(n,G)}$$

and competition value:

$$relComp_C(n,G) = \frac{Comp_C(n,G)}{relC(n,G)}$$

Total robustness dependence and competition value can be used in order to characterize the entire network. Total robustness of a node $n$ with respect to the centrality C in the network $G$ is:

$$TotRob_C(n,G) = \frac{1}{\sum_{i\in N_{|n}} Int_C(i,n,G)}$$

Similarly are defined the total dependence value:

$$TotDep_C(n,G) = \sum_{i\in N_{|n}} Int_C(i,n,G)$$

where

$$Int_C(i,n,G) \geq 0$$

and the total competition value:

$$TotComp_C(n,G) = \sum_{i\in N_{|n}} |Int_C(i,n,G)|$$

where

$$Int_C(i,n,G) \leq 0$$
