# Supplementary material for: Node Interference and Robustness: Performing Virtual Knock-Out Experiments on Biological Networks: The Case of Leukocyte Integrin Activation Network
Source: PLoS One. 2014 Feb 20;9(2):e88938. doi: 10.1371/journal.pone.0088938 (PMC3930642; doi:10.1371/journal.pone.0088938)
Supplement: File S3 — Example of Interference analysis of the Human Kino-Phosphatome network. (PDF) [file pone.0088938.s005.pdf]

Interference in the human kino-phosphatome

In this example the interference analysis is used to drive further lab experiments. We investigated interference in the human kino-phosphatome, a biological signaling interactomic molecular set based on protein-protein interaction that can be abstracted as a complex network of human kinases and phosphatases, in which some proteins are involved in activation and inhibition of other proteins (see suppl. file K-P subnetwork.sif). In previous works we found that Mapk1 and Prkca displays highest centralities values [1] and so are good candidates to control the overall network structure [2, 3].

First ten positive interference value of Mapk1 and Prkca

node	Mapk1 interf.	node	Prkca interf.
PPP1CA	0,0008	PPP2CA	0,0012
PPP2CA	0,0005	MAP3K3	0,0009
CAMK2G	0,0004	AURKA	0,0008
PTEN	0,0004	MAPK9	0,0007
RPS6KA3	0,0003	PTPRC	0,0006
PTPN11	0,0003	PTPN1	0,0004
JAK2	0,0003	CHEK2	0,0004
CDC25C	0,0003	CAMK2G	0,0004
PDPK1	0,0003	PPP1R14A	0,0004
ZAP70	0,0003	STK11	0,0003

Table 1. First ten positive interference values of Mapk1 and Prkca. Only PPP2CA and CAMK2G have high value with both Mapk1 and Prkca

First ten (absolute value) negative interference values of Mapk1 and Prkca

node	Mapk1 interf.	node	Prkca interf.
MAPK3	-0,0034	RPS6KA3	-0,0047
AKT1	-0,0031	GSK3B	-0,0030
MAPK8	-0,0026	MAPK1	-0,0030
PRKCA	-0,0022	PRKDC	-0,0024
CDC2	-0,0017	PRKCB1	-0,0022
SRC	-0,0017	MAPK8	-0,0020
PTK2B	-0,0017	CDC2	-0,0020
FYN	-0,0015	PRKCZ	-0,0019
IGF1R	-0,0013	PTK2B	-0,0019
MAPK14	-0,0011	PRKACA	-0,0017

Table 2. First ten negative interference values for Mapk1 and Prkca. Notice reciprocal high interference between Mapk1 and Prkca

We calculated and compared betweenness interference of Mapk1 and Prkca reported in the two tables above. Interference values are not high, max interference value is around 0.001. This means that the variation of betweenness values is only around 0.1% of the global value and that removing one node does not consistently affect the entire network. This is in agreement with results on robustness and attack tolerance of biological networks [3][4][5]. Notably, only PPP2CA and CAMK2G are present in both tables. This is really rather interesting as it suggests that the effects of removing Mapk1 or Prkca are not identical, showing that Mapk1 and Prkca removal differently affects dintict part of the network. Potentially this means that different functionality in the kino-phosphatome network are affected by the two proteins. Regarding negative interference first ten highest values are higher than for positive interference (as absolute value) indicating that negative interference of Mapk1 and Prkca is more significant than positive interference. MAPK8, CDC2, PTK2B are present in both tables suggesting that they depends on both Mapk1 and Prkca. Interestingly, there is negative interference of Mapk1 with respect to Prkca (interference = -0,0022). This shows that when Mapk1 is inhibited Prkca likely gains a more relevant role in the network. Similarly Prkca has negative interference with respect to Mapk1 (interference= -0,0030): the role of Mapk1 is more relevant if Prkca is not part of the network. This means that Mapk1 and Prkca are “competitors” in the sense that they compete for having a central role in the network but also, according with betweenness meaning, that some functionality of Mapk1 can be replaced by Prkca and conversely some functionality of Prkca can be replaced by

Mapk1: some shortest paths connecting proteins in the network and passing through Mapk1 are replaced by shortest paths passing through Prkca if Mapk1 is removed from the network and similarly for shortest paths passing through Mapk1 when Prkca is removed. In this sense they are "partners" on maintaining some functionality of the network. Finally, observing both positive and negative interference,we can observe that Mapk1 has positive interference with respect to RPS6KA3 and that Prkca has negative interference on the same protein. So, when Mapk1 is present in the network then RPS6KA3 has a more central role and, in contrast, when Prkca is part of the network RPS6KA3 is less relevant: removal of Mapk1 or Prkca have opposite effects on RPS6KA3. These conclusions will be possibly corroborated by experimental evidences by applying current technologies.

[1] Giovanni Scardoni, Michele Petterlini, and Carlo Laudanna. Analyzing biological network parameters with CentiScaPe. *Bioinformatics* , 25(21):2857–2859, 2009.
[2] Paolo Crucitti, Vito Latora, Massimo Marchiori, and Andrea Rapisarda. Error and attack tolerance of complex networks. *Physica A: Statistical Mechanics and its Applications* , 340(1-3):388 – 394, 2004. News and Expectations in Thermostatistics.
[3] Reka Albert, Hawoong Jeong, and Albert-Laszlo Barabasi. Error and attack tolerance of complex networks. *Nature* , 406(6794):378–382, July 2000.
[4] Albert-Laszlo Barabasi and Zoltan N. Oltvai. Network biology: understanding the cell's functional organization. *Nature Reviews Genetics* , 5(2):101–113, February 2004.
[5] H. Jeong, S. P. Mason, A. L. Barabasi, and Z. N. Oltvai. Lethality and centrality in protein networks. *Nature* , 411(6833):41–42, May 2001.
