# Supplementary material for: Node Interference and Robustness: Performing Virtual Knock-Out Experiments on Biological Networks: The Case of Leukocyte Integrin Activation Network
Source: PLoS One. 2014 Feb 20;9(2):e88938. doi: 10.1371/journal.pone.0088938 (PMC3930642; doi:10.1371/journal.pone.0088938)
Supplement: File S5 — Betweenness Interference values for SRC FGR and HCK in the integrin network. (PDF) [file pone.0088938.s007.pdf]

SRC

Node name	Betweenness Interference
JAK2	-2.524990852235719
PIK3R1	-1.5599660565981104
HCK	-1.3906690552669683
PLCG1	-1.362562568347884
RHOA	-1.237850371058033
RAP1A	-1.159558500450716
PRKAB1	-0.9902727886822724
PIK3R2	-0.9139552336462247
TLN1	-0.8948587187419164
SYK	-0.7833395891825452
RAC1	-0.6370612157916327
PLD1	-0.5231072374293726
APBB1IP	-0.5103881447186178
DOCK2	-0.440985285760262…
PKD1	-0.339560136021370…
FYB	-0.333268324517664…
PIK3CB	-0.297050982351942…
STK4	-0.275930931919874
PLCG2	-0.2608500464848649
VAV1	-0.241803734829977…
RASGRP1	-0.222878583158521…
PRKAA2	-0.2078737396221646
CDC42	-0.1900811311531586
PIK3CA	-0.147239667878356…
PIK3CG	-0.1339870358946822
PRKAR2A	-0.133069647567669
JAK3	-0.127433620844089…
PRKCZ	-0.109589382942669…
RASSF5	-0.089472947755330…
PRKACA	-0.054500824871116…
PIK3C2B	-0.046803788944661…
PLCE1	-0.012489384145393…
PIK3CD	-0.006201087999155…
FGR	-0.005294661660315…
CYTH1	0.0
ILK	0.0
PIK3AP1	0.0
PIK3R3	0.0
PIK3R5	0.0
PLCB4	0.0
PRKAG1	0.0
PRKAG2	0.0
PRKAG3	0.0
PRKAR1A	0.0
PRKAR1B	0.0
PRKAR2B	0.0
RHOH	0.0
SWAP70	0.0
PLCB2	0.03851419593023908
PLCB1	0.04278790028660495
ACTN1	0.04584362075749149
PIP5K1C	0.0595645161967078…
PIK3C2A	0.12787149383472318
ARF1	0.15447242877581502
PRKAA1	0.1823947120906637
HRAS	0.22529616680962405
ARF6	0.2329342221425128
SKAP1	0.3670646000490416
PRKAB2	0.4157759151038982
PRKACB	0.6239424752339793

FGR

Node name	Betweenness Interference
PIK3R1	-0.216861672891162…
RAC1	-0.191096877774699…
PRKAB1	-0.118290138496801…
RAP1A	-0.096852539218857…
RHOA	-0.085970355416398…
PIK3CG	-0.073435897186503…
PIK3CA	-0.072155047955866…
JAK2	-0.0695776661840406
HRAS	-0.065133723628778…
PRKACA	-0.061147114390187…
PRKAB2	-0.045417547476272…
TLN1	-0.036639972386077…
ARF1	-0.033970625226207…
PLCG1	-0.032450957757190…
PRKCZ	-0.030963548747549…
FYB	-0.030661416575697…
PLCB1	-0.027552151501479…
PRKAA2	-0.027078155216227…
PLD1	-0.025525393733749…
PRKACB	-0.024051498479528…
ARF6	-0.023266990069435…
VAV1	-0.020434068293418…
PIK3R2	-0.016573913566915…
CDC42	-0.015737994597473…
PLCB2	-0.015624976517509…
PIK3CB	-0.014073313791130…
APBB1IP	-0.012556199123835…
RASSF5	-0.009786152212086…
PRKAR2A	-0.006904348834885…
DOCK2	-0.006504385169795…
JAK3	-0.005311682699157…
RASGRP1	-0.002893561337457…
PIK3CD	-0.001440041855679…
PIK3C2B	-0.001436003403842…
PIP5K1C	-9.409151764910861…
PLCE1	-6.285689056358366…
CYTH1	0.0
ILK	0.0
PIK3AP1	0.0
PIK3R3	0.0
PIK3R5	0.0
PLCB4	0.0
PRKAG1	0.0
PRKAG2	0.0
PRKAG3	0.0
PRKAR1A	0.0
PRKAR1B	0.0
PRKAR2B	0.0
RHOH	0.0
SWAP70	0.0
PKD1	0.0014903828899018…
ACTN1	0.0028325360975789…
STK4	0.0064180009037415…
PLCG2	0.0103966134283544…
SKAP1	0.02806377514069569
PIK3C2A	0.05071455464026736
PRKAA1	0.16595124689854845
HCK	0.1740431085669858
SYK	0.27682485903013365
SRC	0.7811314214776122

HCK

Node name	Betweenness Interference
SRC	-0.456151822166068…
SYK	-0.3364468748885292
RAC1	-0.255364533243477…
JAK2	-0.128757199475002…
PIK3CA	-0.117135365269513…
PIK3CG	-0.098763818737124…
RHOA	-0.097523506545377
PRKAA1	-0.085001021188966…
PRKACA	-0.065040355932069…
RAP1A	-0.056974304798433…
PRKAA2	-0.054630613195357…
HRAS	-0.053605673950681…
FYB	-0.053195803821093…
PLCB2	-0.027367209815239…
ARF6	-0.024073514012643…
PRKCZ	-0.021701768403180…
PIK3C2A	-0.017515497425868
ARF1	-0.014763929974419…
PKD1	-0.013153684743632…
TLN1	-0.012905856613317…
PIP5K1C	-0.011232096525893…
APBB1IP	-0.010588841974139…
PIK3C2B	-0.010094689315718…
FGR	-0.008710866176715…
SKAP1	-0.008486491139065…
ACTN1	-0.008448725159759…
RASSF5	-0.008098447513040…
PRKACB	-0.006776070570624…
STK4	-0.006463778485583…
PRKAR2A	-0.006394170857010…
PLD1	-0.005962420994407…
DOCK2	-0.005854325055539…
PIK3CD	-0.002738247910865…
RASGRP1	-0.002499297546402…
PIK3R1	-0.002427936922503…
PLCE1	-4.636447025595618…
CYTH1	0.0
ILK	0.0
PIK3AP1	0.0
PIK3R3	0.0
PIK3R5	0.0
PLCB4	0.0
PRKAG1	0.0
PRKAG2	0.0
PRKAG3	0.0
PRKAR1A	0.0
PRKAR1B	0.0
PRKAR2B	0.0
RHOH	0.0
SWAP70	0.0
PIK3R2	0.0059967554172837…
PLCB1	0.0091471214304250…
PIK3CB	0.0222621989424586…
JAK3	0.0408086571450114…
PRKAB2	0.04429679466667569
PRKAB1	0.0733883715104593
PLCG1	0.08088835046375209
CDC42	0.08549885044891803
VAV1	0.0957525731561586
PLCG2	0.14017654987591188
