# Supplementary material for: Node Interference and Robustness: Performing Virtual Knock-Out Experiments on Biological Networks: The Case of Leukocyte Integrin Activation Network
Source: PLoS One. 2014 Feb 20;9(2):e88938. doi: 10.1371/journal.pone.0088938 (PMC3930642; doi:10.1371/journal.pone.0088938)
Supplement: File S6 — Betweenness Interference values for JAK2 in the integrin network. (PDF) [file pone.0088938.s008.pdf]

**NETWORK**	**IntegrinActivationNetwo...**
**SELECTED NODE**	**JAK2**

Node name	Betweenness Interference
SRC	-1.337
HRAS	-0.303
RHOA	-0.283
SYK	-0.258
PRKACB	-0.161
HCK	-0.157
PIK3CB	-0.114
PRKACA	-0.113
PRKAA2	-0.106
PIK3R1	-0.076
PIK3R2	-0.072
RAC1	-0.069
CDC42	-0.068
TLN1	-0.066
FYB	-0.065
PRKAB1	-0.052
SKAP1	-0.046
PIK3CG	-0.036
STK4	-0.03
VAV1	-0.029
PLCB1	-0.028
PRKCZ	-0.028
RASSF5	-0.024
APBB1IP	-0.02
PIK3C2A	-0.02
PLD1	-0.018
PIP5K1C	-0.012
ACTN1	-0.01
DOCK2	-0.008
PRKAR2A	-0.006
ARF6	-0.003
RASGRP1	-0.003
ARF1	-0.001
FGR	-0.001
PIK3C2B	-0.001
PIK3CD	-0.001
PLCE1	-0.001
CYTH1	0.0
ILK	0.0
PIK3AP1	0.0
PIK3R3	0.0
PIK3R5	0.0
PLCB4	0.0
PRKAG1	0.0
PRKAG2	0.0
PRKAG3	0.0
PRKAR1A	0.0
PRKAR1B	0.0
PRKAR2B	0.0
RHOH	0.0
SWAP70	0.0
JAK3	0.001
PLCB2	0.012
PLCG1	0.018
PIK3CA	0.026
PLCG2	0.032
PKD1	0.08
PRKAB2	0.095
PRKAA1	0.13
RAP1A	0.401
