# Supplementary material for: Node Interference and Robustness: Performing Virtual Knock-Out Experiments on Biological Networks: The Case of Leukocyte Integrin Activation Network
Source: PLoS One. 2014 Feb 20;9(2):e88938. doi: 10.1371/journal.pone.0088938 (PMC3930642; doi:10.1371/journal.pone.0088938)
Supplement: File S7 — Interference definition extended to subset of nodes. (PDF) [file pone.0088938.s009.pdf]

1 Suppl. File S7: Definitions extended to subsets of nodes

Given a subset S of the network nodes N ($S \in N$) and a centrality measure C, the interference of the set of nodes S with respect to node n in the network G is:

$$Int_C(S, n, G) = \frac{C(G, n)}{\sum_{j \in N} C(G, n)} - \frac{C(G \backslash S, n)}{\sum_{j \in N} C(G \backslash S, n)}$$

Max interference, global interference and mean interference are similarly defined. Besides the definitions can be easily adapted if we are interested in removing or adding one or more edges in a network.
